# Synthesis, Characterization and Biological Evaluation of Magnolol and Honokiol Derivatives with 1,3,5-Triazine of Metformin Cyclization

**DOI:** 10.3390/molecules25245779

**Published:** 2020-12-08

**Authors:** Cui Ren, Juanxia Wang, Youzhen Tan, Mingxin Guo, Jieqing Guo, Ying Liu, Xia Wu, Yifan Feng

**Affiliations:** New Drug Research and Development Center, Guangdong Pharmaceutical University, Guangzhou 510006, China; rrencui@163.com (C.R.); 15698959310@163.com (J.W.); tanyouzhenprimrose@126.com (Y.T.); guomingxin94@126.com (M.G.); gjq921493@163.com (J.G.); ly953403517@163.com (Y.L.)

**Keywords:** magnolol, honokiol, metformin, chemical synthesis, biological activity

## Abstract

Herein, we sought to evaluate the contribution of the 1,3,5-triazine ring through the metformin cyclization unit to the biological activity of magnolol and honokiol-conjugates. One of the phenolic OH groups of magnolol or honokiol was replaced by a 1,3,5-triazine ring to further explore their synthesis and medicinal versatility. In this study, a robust procedure of three steps was adopted for the synthesis of magnolol and honokiol derivatives by alkylation of potassium carbonate with a 1,3,5-triazine ring. To our knowledge, this is the first report to connect one of the phenolic OH positions of magnolol or honokiol to a 1,3,5-triazine ring cyclized by metformin. The structural characterization of three new compounds was carried out via spectroscopic techniques, i.e., ^13^C NMR, ^1^H NMR, and HRMS. Surprisingly, these compounds showed no cytotoxicity against RAW 264.7 macrophages but significantly inhibited the proliferation of MCF-7 (human breast cancer cells), HepG2 (human hepatoma cells), A549 (human lung carcinoma cells), and BxPC-3 (human pancreatic carcinoma cells) tumor cell lines. Furthermore, the compounds also significantly inhibited the release of inflammatory cytokines, including nitric oxide (NO), tumor necrosis factor-α (TNF-α), and interleukin-1β (IL-1β) in the lipopolysaccharide (LPS)-activated mouse cells (RAW 264.7). Among them, compound 2 demonstrated promising broad-spectrum antiproliferative potential with half inhibitory concentration (IC_50_) values ranging from 5.57 to 8.74 µM and it significantly decreased caspase-3 and Bcl-2 expression in HepG2 cells. These interesting findings show that derivatization of magnolol and honokiol with 1,3,5-triazine affects and modulates their biological properties.

## 1. Introduction

*Magnolia officinalis* is a flowering plant that is native to China, South Korea, and Japan. The root and stem bark of this plant are enriched with two bioactive constituents, magnolol and honokiol, with the IUPAC names of 1,5,5′-diallyl-2,2′-dihydroxybiphenyl and 2,5,5′-diallyl-2,4′-dihydroxybiphenyl, respectively. These compounds are the isomers of hydroxylated biphenyls [1]. These bioactive compounds are known to possess therapeutic properties such as anti-oxidative, anti-microbial, anti-atherosclerosis, anti-inflammatory, and anticancer properties [2,3,4,5,6,7]. 1,1-Dimethylbiguanide, also known as glucophage or metformin, is classified in the class of organic compounds called biguanides. Metformin is isolated from *Galega officinalis* (French Lilac) and is a widely prescribed medication against type 2 diabetes mellitus (T2DM). In the 1950s, its usefulness as an oral medication against hyperglycemia became widely known [8]. According to the recently reported studies (basic and clinical studies), this anti-diabetic agent is also known for a variety of other significant bioactivities, such as activity against aging, cancer, and inflammation [9,10,11,12].

Based on a review of the latest improvements in metformin, magnolol, and honokiol structure modifications, various compounds have been efficiently synthesized via their derivatization process. Margherita Maioli synthesized (in 2018) some magnolol and honokiol derivatives and found that derivatization of honokiol with butyrate ester at one of its phenol-OH groups significantly enhanced its in vitro antiproliferative activity against hepatocellular carcinoma [13]. In the same year (2018), Tong-Hua Yang synthesized 23 compounds (reprivatized from magnolol) and found outstanding antidepressant effects both in vitro and in vivo, particularly, when magnolol included glucose attached to one of the phenolic hydroxyl groups [14]. In this year, Yang Hu also designed and synthesized six derivatives of honokiol and magnolol. These derivatives with a double bond and an OH group exhibited outstanding inhibitory potential against *Saprolegnia parasitica* growth [15]. In 2012, Minsoo Koh synthesized a novel derivative of metformin and metformin salts, i.e., metformin pregabalin salt, which showed highly potent inhibition against Hs578T triple-negative breast cancer cell line growth and invasive ability in comparison to the parent metformin [16].

**Figure 1:**
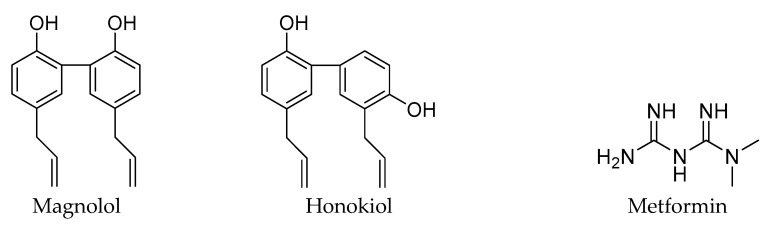

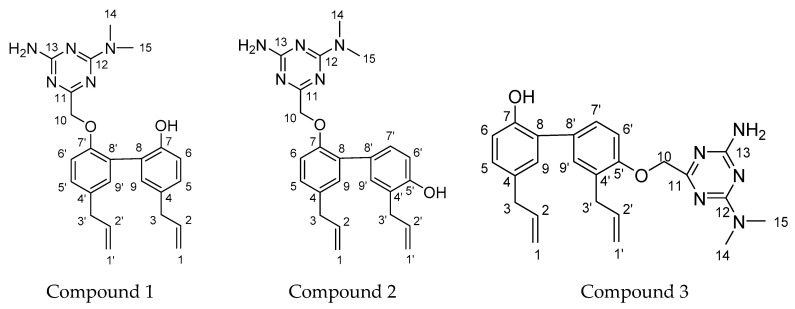
The structures of magnolol, honokiol, metformin, and compound 1, 2, 3.

However, the aforementioned reports on magnolol, honokiol, and metformin derivatives are paving the way for individual structural transformations. Therefore, based on the research of previously reported literature and our laboratory experiments, we identified that the biological activity of magnolol or honokiol could be increased to the desired level by introducing a pharmacophore into one of its phenolic OH groups and exposing the other. In our previous study, we synthesized three novel target products, which combined two pharmacophores from berberine, magnolol, and metformin. All of the products showed remarkable anti-inflammatory and antidiabetic effects in INS-1 cells and RAW 264.7 cells [17]. This study is part of our continuous research on the chemistry of metformin, magnolol, and honokiol, and refers to our previous synthetic strategies that we adopted in our laboratory. In addition, the structurally diverse schemes of compounds 1, 2, and 3 (Figure 1) encouraged us to undertake further studies in this field. In this report, we designed the synthesis of three magnolol and honokiol derivatives with 1,3,5-triazine of metformin cyclization via suitable chemical transformation. Structural elucidations of the obtained compounds were conducted via ^13^C NMR, ^1^H NMR, and HRESIMS spectral analysis. Then, the underlined obtained compounds were screened against inflammation and cancer cells, i.e., RAW 264.7, MCF-7, HepG2, A549, and BxPC-3 cell proliferation. The cytotoxic and anticancer activities of these derivatives were checked via 3-(4,5-dimethylthiazol-2-yl)-2,5-diphenyltetrazolium bromide (MTT)-based experiments [18]. In the existing study, we also evaluated the underlined compounds for their suppression activity of the pro-inflammatory mediators including IL-1β, TNF-α, and NO in the LPS-activated RAW 264.7 macrophage cells. The results of this experiment revealed that compound 2 possesses potent anti-inflammatory and anti-cancer effects. Based on this result, compound 2 proceeded to evaluation of its effect on the regulation of the Bcl-2 and caspase-3 expression level in HepG2 cells via immunoblotting.

## 2. Results and Discussion

### 2.1. Chemistry

**Scheme 1:**
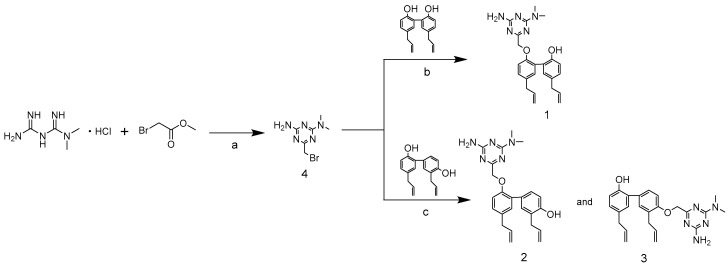
Depiction of the synthetic pathway of compound-1, -2 and -3. a: CH_3_ONa, EtOH, 0 °C, 1 h; b: K_2_CO_3_, EtOH, 75 °C, 6 h; c: K_2_CO_3_, CH_3_OH, 60 °C, 5 h.

Scheme 1 represents the synthetic pathways of compound 1, 2, and 3, respectively [17]. First, the cyclization of metformin hydrochloride and methyl bromoacetate, which was catalyzed by sodium methoxide, was performed in ethanol at 0 °C for 1 h and then the intermediate 1,3,5-triazine derivative (compound 4) was obtained. Subsequently, the alkylation of magnolol on phenolic hydroxyl (OH) groups with 1,3,5-triazine derivatives was catalyzed by potassium carbonate in ethanol solution at 75 °C for 6 h, which resulted in the final magnolol derivatives (compound 1). For compound 1, the two phenol OH groups of magnolol have greater steric hindrance with each other and only one of the OH groups can be connected to a 1,3,5-triazine ring. The alkylation of honokiol on phenolic hydroxyl (OH) groups with 1,3,5-triazine derivatives was catalyzed by potassium carbonate in methanol solution at 60 °C for 5 h, which resulted in the final honokiol derivatives (compounds 2 and 3). For compounds 2 and 3, one of the phenol OH groups of honokiol was introduced to a 1,3,5-triazine ring, the activity of the other OH group was reduced, and it became very difficult to connect to the other 1,3,5-triazine ring. Therefore, only one 1,3,5-triazine ring could be introduced to the OH groups of honokiol. In addition, the activity of the 5′-OH of honokiol was greater than the 7-OH, and the yield of compound 3 (31%) was more than compound 2 (12%). Post crystallization and purification, the structures of those products were firmly established by HRMS, ^1^H NMR, and ^13^C NMR spectra.

### 2.2. Biological Evaluation

#### 2.2.1. The Inhibitory Effect of the Compounds on LPS-Activated NO, TNF-α, and IL-1β Release

**Figure 2:**
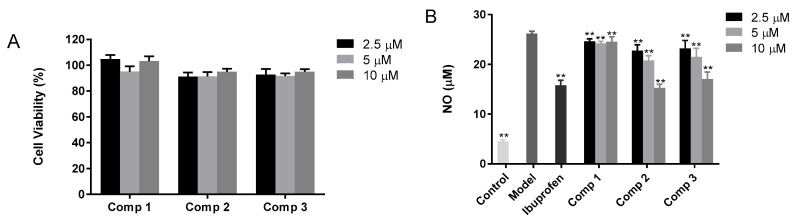

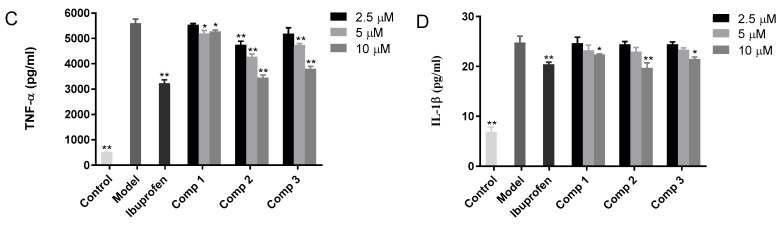
(**A**) The cytotoxicity of the compounds against mouse macrophage cells (RAW 264.7). (**B**) The effects of the compound on the release of NO from RAW 264.7 cells (LPS-activated). (**C**) The effect of the compound on the release of TNF-α from RAW 264.7 cells (LPS-activated), while (**D**) indicates the effect of the compound on the release of IL-1β from RAW 264.7 cells (LPS-activated). The stimulation of RAW 264.7 cells was carried out with 2 µg per mL of LPS in the presence of the compounds, while the compounds were not present in the control. Griess and ELISA methods were employed for the evaluation of the secreted level of NO, TNF-α, and IL-1β in the supernatants, post overnight stimulation of LPS. * *p* < 0.05, ** *p* < 0.01 was compared to the model.

First, the MTT-based experiment was carried out in order to evaluate the cytotoxicity of compound 1, 2, and 3 in the LPS-activated mouse cells (RAW 264.7). Then the activity of the underlined compounds was evaluated against inflammation in these cells. As shown in Figure 2A, the acquired data suggested that up to 10.00 µM concentration, the compounds were non-cytotoxic. Therefore, for the investigation of the anti-inflammatory effect of the compounds, the assays were conducted at a concentration of 2.50, 5.00, and 10.00 µM in RAW 264.7 mouse cells (LPS-induced). To assess the capability of the underlined compounds in suppressing NO, TNF-α, and IL-1β release from macrophage RAW 264.7 cells (LPS-induced), the medium containing mouse RAW 264.7 cells was incubated with LPS and then the effect of the compounds on LPS-induced release of NO, TNF-α, and IL-1β was measured. In particular, 2 µg per mL of LPS stimulated the RAW 264.7 mouse cells in the presence of the respective compounds at 2.50, 5.00, and 10.00 µM, and the synthesis of NO was checked via the Griess method, which investigated the accumulation of nitrite in a culture medium. The obtained results revealed that ibuprofen suppressed the synthesis of NO by 47.94%, compound 1 at 2.50, 5.00, and 10.00 µM inhibited NO synthesis by 7.31, 9.23, and 7.62%, respectively, compound 2 at 2.50, 5.00, and 10.00 µM inhibited the synthesis of NO by 15.81, 24.89 and 50.39%, respectively, while compound 3 at 2.50, 5.00 and 10.00 µM suppressed NO synthesis by 13.80, 21.49 and 42.08%, respectively, as depicted in Figure 2B. The ELISA kits were used for the identification of the inhibitory effect of TNF-α release by the compounds in LPS (2.00 µg/mL)-activated RAW 264.7 cells. The obtained results revealed that ibuprofen suppressed the production of TNF-α by 46.56%, compound 1 at 2.50 µM, 5.00 µM and10 µM inhibited TNF-α production by 1.18, 7.93, and 6.46%, respectively, compound 2 at 2.50, 5.00, and 10.00 µM inhibited TNF-α production by 16.69, 26.03 and 42.11%, respectively, while compound 3 at 2.50, 5.00 and 10.00 µM suppressed the synthesis of TNF-α by 8.18, 16.89 and 35.40%, respectively, as indicated in Figure 2C. The inhibitory effect of IL-1β release by compounds in LPS (2.00 µg/mL)-activated RAW 264.7 cells was assessed via ELISA kits. The acquired results demonstrated that ibuprofen inhibited the production of IL-1β by 24.51%, compound 1 at 2.50, 5.00 and 10.00 µM inhibited the production of IL-1β by 0.86, 8.72, and 13.63%, respectively, compound 2 at 2.50, 5.00 and 10.00 µM inhibited IL-1β production by 1.97, 10.30 and 28.46%, respectively, while compound 3 at 2.50, 5.00 and 10.00 µM inhibited IL-1β production by 1.92, 7.93 and 18.53%, respectively, as depicted in Figure 2D.

In summary, all compounds significantly decreased the release of the inflammatory cytokines NO, TNF-α, and IL-1β from macrophage RAW 264.7 cells compared with the model group in a dose-dependent manner. Furthermore, the inhibitory effects of compounds 2 and 3 were comparable to ibuprofen at high concentrations. These findings implicated that the anti-inflammatory activity of magnolol and honokiol will be increased if they combine a 1,3,5-triazine at one of the phenol-OH groups.

#### 2.2.2. The Inhibitory Potential of Compounds against the Proliferative Ability of Various Human Carcinoma Cells

To determine the inhibitory potency of acquired compounds against the proliferation of various human carcinoma cells, MCF-7, HepG2, A549, and BxPC-3 cells were exposed to various concentrations of obtained compounds for 2 days. As shown in Table 1, compounds 1, 2, and 3 showed comparatively low micromole IC_50_ values compared to magnolol and honokiol. Among them, compound 2 demonstrated promising and broad-spectrum antiproliferative activity with IC_50_ values ranging from 5.57 to 8.74 µM. Hence, this suggests that these compounds might be potential broad-spectrum antitumor agents for human cancer treatment.

#### 2.2.3. Compound 2 Inhibited Bcl-2 and Caspase-3 Protein Expression in HepG2 Cells

**Figure 3:**
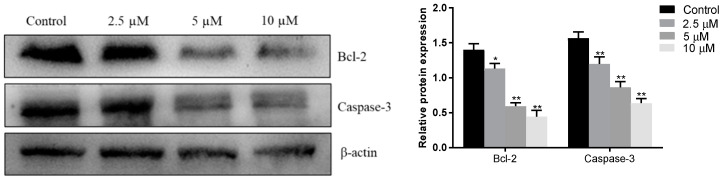
The impact of compound 2 on the expression level of Bcl-2 and caspase-3 in HepG2 cells. Each experiment was performed thricely in an independent manner and the obtained results are given as mean ± SEM. * *p* < 0.05, ** *p*-value < 0.01 compared to the control.

In this study, HepG2 cells were selected as target cells for further study, as depicted in Figure 3. The obtained results of the MTT assay indicated that compound 2 (dose-dependent) considerably suppressed HepG2 cell proliferation, as shown in Table 1. To identify whether the inhibition of HepG2 cell proliferation (induced via compound 2) was correlated with Bcl-2 and caspase-3, immunoblotting was conducted to evaluate Bcl-2 and caspase-3 expression in HepG2 cells post-incubation with compound 2 at various concentrations, i.e., 2.5, 5, and 10 μM. As depicted in Figure 3, compound 2 exposure reduces the dose-dependent expression of Bcl-2 and caspase-3. These findings suggest that compound 2 treatment exhibited obvious anti-cancer efficacy in HepG2 cells and its underlying mechanism could be associated with its decreasing effect on the expression of Bcl-2 and caspase-3 proteins.

## 3. Experimental Section

### 3.1. General Information

To determine the melting point of each compound, the Beijing Tech X-6 capillary melting point apparatus (China) was employed. An AVANCE-III HD spectrometer (Bruker, Karlsruhe, Germany) was used to acquire the ^13^C-NMR and 1H-NMR spectral data (Supplementary Materials). DMSO or CH_3_OH-*d*_6_ was utilized as the solvent, and tetramethylsilane as an internal reference solvent. Chemical shifts (δ) and coupling constants (*J*) are given in ppm and Hz, respectively. Abbreviations for signal coupling are as follows: s, singlet; d, doublet; m, multiplet. Mass spectra analysis was carried out on a SCIEX X500R QTOF mass spectrometer equipped with a twin spray electrospray ionization (ESI) interface and controlled by X500R SCIEX OS v1.4 solftware (SCIEX, CA, USA). The HPLC analyses of the obtained compounds were carried out via HPLC-e2695 (Waters, MA, USA) with a 5.00 µm C18 column (200 × 4.6 mm). Purifications by flash chromatography were performed using Merck 107,736 silica gel 60 (particle size 0.040–0.062 mm) using an ethyl acetate-dichloromethane solvent system. The reactions were magnetically stirred and monitored by analytical thin-layer chromatography on silica gel 60 F_254_ (Merck, Darmstadt, Germany) and UPLC-MS on a Waters UPLC-Q-TOF-MS (Waters, MA, USA). All reagents were purchased from commercial suppliers without prior purification.

### 3.2. General Synthetic Procedure for Compounds

#### 3.2.1. Compound 1

5,5′-diallyl-2′-((4-amino-6-(dimethylamino)-1,3,5-triazin-2-yl)methoxy)-[1,1′-biphenyl]-2-ol (1). Magnolol (2.00 g, 0.0075 M) was reacted with compound-4 (2.30 g, 0.01 M) and potassium carbonate (anhydrous, 1.4 g, 0.01 M) in 50.00 mL absolute EtOH at 75 °C for 6 h. Then, the reaction-mixture was placed in a refrigerator in order to obtain the crystals of the desired compound, i.e., compound 1, followed by washing with MeOH (anhydrous) and drying in a vacuum. White powder; M.P. 213–214 °C; Yield: 58.2%; ESI-MS *m*/*z* calculated for C_24_H_28_N_5_O_2_ [M + H]^+^ 418.2237, found 418.2217; ^1^H-NMR (500 MHz, DMSO-*d*_6_) δ ppm: 2.96 (6H, d, *J* = 20 Hz, H-14, H-15), 3.27–3.32 (4H, m, H-3, H-3′), 4.84 (2H, s, H-10), 4.98–5.07 (4H, m, H-1, H-1′), 5.89–5.98 (2H, m, H-2, H-2′), 6.96 (2H, d, *J* = 5.0 Hz, H-6, H-6′), 6.99 (2H, d, *J* = 5.0 Hz, H-9, H-9′), 7.03 (2H, d, *J* = 5.0 Hz, H-5, H-5′), 6.81 (2H, d, *J* = 5.0 Hz, -NH_2_), 8.74 (1H, -OH); ^13^C-NMR (126 MHz, DMSO-*d*_6_) δ = 172.66 (C, C-11), 166.13 (C, C-13), 164.80 (C, C-12), 153.41 (C, C-7′), 152.79 (C, C-7), 138.23 (C, C-2′), 137.97 (C, C-2), 131.65 (C, C-4), 131.54 (C, C-9′), 131.20 (C, C-9), 130.13 (C, C-4′), 128.24 (C, C-5′), 128.18 (C, C-5), 127.37 (C, C-8′), 125.81 (C, C-8), 116.56 (C, C-6), 115.50 (C, C-1′), 115.21 (C, C-1), 112.11 (C, C-6′), 68.67 (C, C-10), 38.71 (C, C-3′), 38.58 (C, C-3), 35.64 (C, C-14, C-15).

#### 3.2.2. Compound-2 and -3

3,5′-diallyl-2′-((4-amino-6-(dimethylamino)-1,3,5-triazin-2-yl)methoxy)-[1,1′-biphenyl]-4-ol (2); 3′,5-diallyl-4′-((4-amino-6-(dimethylamino)-1,3,5-triazin-2-yl)methoxy)-[1,1′-biphenyl]-2-ol (3); Honokiol (2.00 g, 0.0075 M) was reacted with compound-4 (2.30 g, 0.01 M) and potassium carbonate (anhydrous, 1.4 g, 0.01 M) in MeOH (anhydrous) at 60 °C for 5 h. Filtration of the reaction-mixture was performed, followed by evaporation of the solvent until it formed a yellow viscous solid. Next, purification of the obtained yellow viscous solid was done via flash-chromatography using dichloromethane and ethyl acetate (7:3) as the eluent. Compound-2: yellow solid; M.P. 231–232 °C; Yield: 12%; ESI-MS *m*/*z* calculated for C_24_H_28_N_5_O_2_ [M + H]^+^ 418.2237, found 418.2216; 1H-NMR (500 MHz, CH_3_OH-*d*_6_) δ ppm: 3.07 (6H, s, H-14, H-15), 3.31–3.36 (4H, m, H-3, H-3′), 4.72 (2H, s, H-10), 4.94–5.07 (4H, m, H-1, H-1′), 5.93–6.00 (2H, m, H-2, H-2′), 6.76 (1H, d, *J* = 5.0 Hz, H-6), 6.89 (1H, d, *J* = 5.0 Hz, H-6′), 6.99 (1H, d, *J* = 5.0 Hz, H-9), 7.07 (1H, d, *J* = 5.0 Hz, H-9′), 7.29 (1H, d, *J* = 5.0 Hz, H-5), 7.31 (1H, s, H-7′); ^13^C-NMR (126 MHz, CH_3_OH-*d*_6_) δ = 174.69 (C, C-11), 168.11 (C, C-13), 166.67 (C, C-12), 155.53 (C, C-7), 155.35 (C, C-5′), 139.44 (C, C-2), 138.68 (C, C-2′), 134.52 (C, C-4), 132.75 (C, C-4′), 132.47 (C, C-9), 131.89 (C, C-9′), 131.34 (C, C-8), 129.61 (C, C-6), 128.79 (C, C-6′), 127.24 (C, C-8′), 115.80 (C, C-7′), 115.58 (C, C-5), 115.43 (C, C-1), 115.24 (C, C-1′), 71.84 (C, C-10), 40.58 (C, C-3′), 36.65 (C, C-14, C-15), 35.42 (C, C-3). Compound-3: white solid; M.P. 256–257 °C; Yield: 31%; ESI-MS *m*/*z* calculated for C_24_H_28_N_5_O_2_ [M + H]^+^ 418.2237, found 418.2216; ^1^H-NMR (500 MHz, CH_3_OH-*d*_6_) δ ppm: 3.09 (6H, s, H-14, H-15), 3.27–3.31 (2H, m, H-3), 3.49 (2H, d, *J* = 5.0 Hz, H-3′), 4.85 (2H, s, H-10), 4.98–5.10 (4H, m, H-1, H-1′), 5.90–6.12 (2H, m, H-2, H-2′), 6.77 (1H, d, *J* = 10.0 Hz, H-6), 6.89 (1H, d, *J* = 10.0 Hz, H-6′), 6.90–6.92 (1H, m, H-9), 6.99 (1H, d, *J* = 5.0 Hz, H-9′), 7.28-7.30 (1H, m, H-5), 7.31 (1H, d, *J* = 5.0 Hz, H-7′); ^13^C-NMR (126 MHz, CH_3_OH -*d*_6_) δ = 174.76 (C, C-11), 168.14 (C, C-13), 166.67 (C, C-12), 156.73 (C, C-5′), 153.53 (C, C-7), 139.66 (C, C-2′), 138.70 (C, C-2), 133.18 (C, C-4′), 132.66 (C, C-4), 132.03 (C, C-9′), 131.74 (C, C-9), 129.87 (C, C-8′), 129.73 (C, C-8), 129.26 (C, C-6′), 129.14 (C, C-6), 117.10 (C, C-7′), 115.75 (C, C-1′), 115.57 (C, C-1), 112.98 (C, C-5), 71.26 (C, C-10), 40.56 (C, C-3′), 36.64 (C, C-14, C-15), 35.69 (C, C-3).

#### 3.2.3. Compound-4

6-(bromomethyl)-N^2^, N^2^-dimethyl-1,3,5-triazine-2,4-diamine (4). Metformin hydrochloride (1.7 g, 0.01 M) and sodium methoxide (2.00 mL, 0.01M) were mixed in absolute ethanol (50 mL), followed by mixing in a solvent at approximately 25 °C for 0.50 h. Then, methyl bromoacetate (1.20 mL, 0.01 M) was mixed (drop by drop) to a cyclization reaction with metformin, followed by stirring for 1 h (at 0 °C). Next, the reaction mixture was filtered and a white solid was obtained, with a yield of 89.45%; M.P. 132-134 °C; ESI-MS *m*/*z* 232.7455 M^+^; ^1^H-NMR (500 MHz, DMSO-*d*_6_) δ ppm: 3.03 (6H, d, *J* = 15 Hz, H-5, H-6), 4.26 (2H, s, H-1), 6.92 (2H, s, -NH_2_); ^13^C-NMR (126 MHz, DMSO-*d*_6_) δ = 171.85, 166.77, 165.23, 46.64, 35.62. 

### 3.3. HPLC Analysis of Obtained Compounds

The quantitative analysis of the obtained compounds was done via HPLC, employing empower chromatography software. The analysis of each sample was conducted using a reverse-phase C18 column (5.00 µm, 250 × 4.6 mm) at approximately 25 °C. The analyte flow-rate was 1.00 mL per min. For the compounds, the mobile phases-A comprised of 0.20% phosphoric acid and 99.80 % H_2_O (*v*/*v*). Acetonitrile was utilized as a mobile phase-B. For compound-1, the gradient elution was given as: initially, 0 to 15 min, 90% A and 10% B; 15–30 min, 25% A and 75% B. For compound-2 and -3, the gradient elution was given as: in the beginning, 0 to 10 min, 90% A and 10% B; 10 to 20 min, 55% A and 45% B; 20 to 30 min, 20% A and 80% B. For compound 4 the gradient elution was as follows: 90% A and 10% B. The flow-rate was 1.00 mL per min. The analyte volume (injection) was 10.00 µL. The UV absorbance was recorded at a single wavelength, 254 nm.

### 3.4. Biology

#### 3.4.1. The Evaluation of Compound Cytotoxicity against RAW 264.7, MCF-7, HepG2, A549, and BxPC-3 Cells

The RAW 264.7, MCF-7, HepG2, A549, and BxPC-3 cell lines were purchased from ATCC (USA). To examine the effect of compound-1, -2, and -3 on cell (RAW 264.7, MCF-7, HepG2, A549, and BxPC-3 cells) viability and proliferation, a MTT-based assay was used, as suggested by the manufacturer’s protocol. Cells (1 × 10^4^ cells) were grown into each well of 96-well microplates, followed by overnight incubation in 5% CO_2_ (at 37 °C). On the following day, the compounds were exposed to the cells at concentrations of 1.25, 2.50, 5.00, 10.00, 20.00, 40.00, 80.00, and 160.00 μM, respectively while in the control, the cells were untreated. Again, the cells were incubated for 2 days. Then the solution of 20.00 μL MTT (0.50 mg/mL) was added to each well. Next, four hrs incubation was carried out at 37 °C. The dissolution of formazan crystals was carried out by the addition of DMSO (150.00 μL). The O.D. was measured at 570 nm via microplate reader (Thermo Fisher, MA, USA). The viability of cells was represented as (A_570nm_ of the treated sample)/(A_570nm_ of control) × 100%. The logit method was used for the measurement of IC_50_ values which is the half-maximal inhibitory concentration of cell viability.

#### 3.4.2. Evaluation of NO Synthesis from LPS-Activated RAW 264.7 Cells

Based on cytotoxicity results of compound 1, 2, and 3 against mouse macrophages, we selected the concentrations on which the survival rate of cells was between 80 to 90% which was dependent on dose. RAW 264.7 cells (5 × 10^5^ cells/mL) were seeded in 96-well plates and then activated with 2 μg per mL of LPS for 60 min. Then, for 24 h the cells were exposed to the medium that consists of various linear concentrations of compounds, i.e., 2.50 μM, 5.00 μM and 10.00 μM. As positive control, ibuprofen (Sigma Inc, Shenzhen, China) was used. Isolated supernatant fractions were treated with the equal volume of Griess reagent (sulfanilamide (1%), naphthylethylenediamine dihydrochloride (0.1%) and phosphoric acid (2%)) and then the incubation was carried out for 10 min at ~25 °C. The O.D. was measured at 546 nm for the quantification of nitrite synthesis and concentrations were identified via standard curve produced with NaNO_2_.

#### 3.4.3. Analysis of TNF-α as well as IL-1β Release from LPS-Activated RAW 264.7 Cells

RAW 264.7 cells (5 × 10^4^ cells) were grown into each well of 96-well plates and then incubated for 24 h at 37 °C. The cells were exposed to compounds 1–3, followed by incubation at 37 °C for 60 min. Then, LPS (2 μg/mL) was mixed, followed by incubation of the cells for 6 h at 37 °C. The TNF- α and IL-1β concentrations were evaluated in each sample via mouse TNF-α and IL-1β ELISA kit (Shanghai Beyotime Biotechnology, Shanghai, China), respectively, as suggested by the manufacturer’s protocol.

#### 3.4.4. Immunoblotting

After diverse treatments, the HepG2 cell washing was carried out thricely with PBS (ice-cold) and the lysis of the underlined cells was carried out in previously cooled lysis buffer provided with PMSF (1%), followed by incubation on ice for 0.5 h. Then, cell centrifugation (12,000× *g*) was carried out at 4 °C (for 15 min). The supernatant was kept at −20 °C. The BCA method was employed for the determination of protein concentration. In each sample, equal amounts of protein were added into each well, which was isolated on SDS-PAGE gel (12%), followed by transfer to PVDF membranes. The blockage of the membrane was carried out by skimmed milk (5%) for 2 h. Tris-buffered saline-Tween20 solution (TBST) was used for washing. The incubation of the membrane was carried out with primary antibodies for 24 h at 4 °C, followed by washing with TBST thricely. Then, membrane incubation was carried out for 2 h with secondary antibodies conjugated with anti-mouse horseradish peroxidase at ~25 °C. An enhanced chemiluminescent substrate was used for the detection of the protein bands. The relevant band’s density was identified and measured via Image Lab v3.0 software in the ChemiDocXRS system (Bio-Rad, Berkeley, CA, USA).

## 4. Conclusions

In conclusion, three novel compounds, i.e., compound 1, 2, and 3, were synthesized, which combined pharmacophores from a 1,3,5-triazine ring. Post synthesis, the evaluation of the underlined compound effect against inflammation and cancerous cells, i.e., RAW 264.7, HepG2, A549, BxPC-3, and MCF-7 cell progression, were carried out in vitro. For the first time, we have reported the underlined activities. Furthermore, this method delivered an economical, safe, and simpler way for the synthesis of 1,3,5-triazine derivatives. Compound 1, 2, and 3 represented considerable anti-inflammatory potency in RAW 264.7 cells, as compared with ibuprofen. Among these compounds, compounds 2 and 3 revealed significant anticancer effects in HepG2, MCF-7, A549, and BxPC-3 cells as compared to the parent compounds, i.e., magnolol and honokiol. While compound-2 revealed the remarkable inhibitory activity on proliferation and considerably lowered the expressions of the Bcl-2 and caspase-3 in HepG2 cells. The obtained results showed that compound 1, 2, and 3 were capable of lowering NO, TNF-α, and IL-1β release and suppressed the growth and development of human carcinoma cell lines. In short, the existing study revealed that compound 1, 2, and 3 have significant activity against inflammation and tumor cells. The introduction of a 1,3,5-triazine ring might enhance the activity, therefore, these results are of great significance for the design, synthesis, and development of novel anti-cancer and anti-inflammatory agents containing a 1,3,5-triazine ring. Furthermore, many pharmacophoric groups were highly compatible with optimized conditions. Therefore, they might be potential targets in medicinal chemistry.

## Figures and Tables

**Table 1:** Antiproliferative activity of compound 1, 2, and 3 against four human cancer cell lines.

Compound	IC50 (µM)			
	MCF-7	HepG2	A549	B × PC-3
Magnolol	64.71 ± 0.73	51.23 ± 0.61	52.11 ± 0.94	74.02 ± 0.33
Honokiol	45.93 ± 0.46	41.84 ± 0.78	33.5 ± 0.48	49.24 ± 1.09
1	24.71 ± 0.58	31.23 ± 0.34	32.11 ± 0.17	34.02 ± 0.75
2	6.63 ± 0.39	5.57 ± 0.26	6.95 ± 0.27	8.74 ± 0.41
3	13.84 ± 0.53	14.42 ±1.31	18.5 ± 0.52	10.31 ± 0.87

## Supplementary Materials

The Supplementary Materials are available online. NMR and HRMS spectra for the target compounds 1, 2 and 3.
