# Supplementary figures and images for: Synthesis, Characterization and Biological Evaluation of Magnolol and Honokiol Derivatives with 1,3,5-Triazine of Metformin Cyclization
Source: Molecules. 2020 Dec 8;25(24):5779. doi: 10.3390/molecules25245779 (PMC7762331; doi:10.3390/molecules25245779)

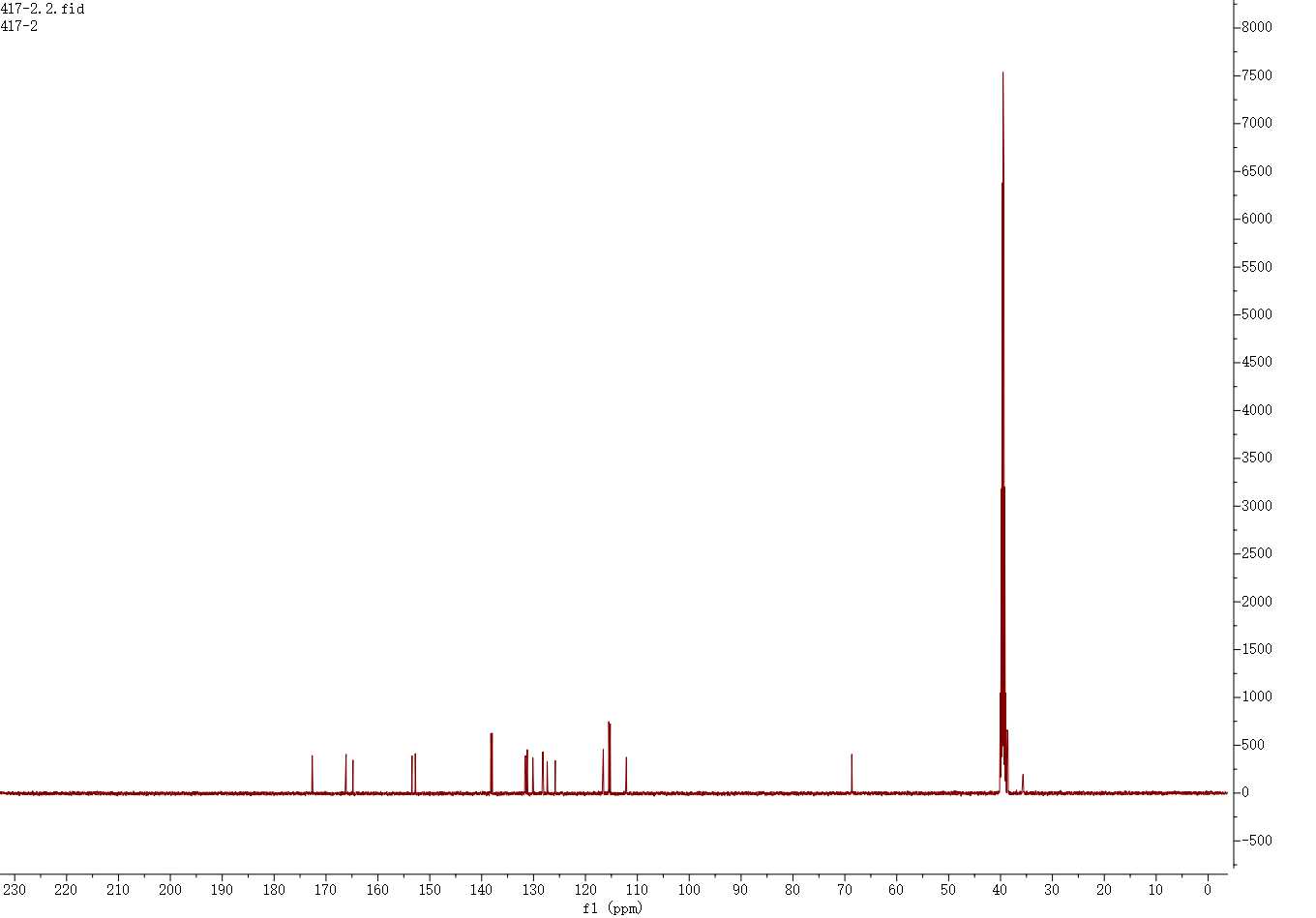

Supplement: Supplementary file 1 [file molecules-25-05779-s001.zip › SI/Compound1_13CNMR.jpg]

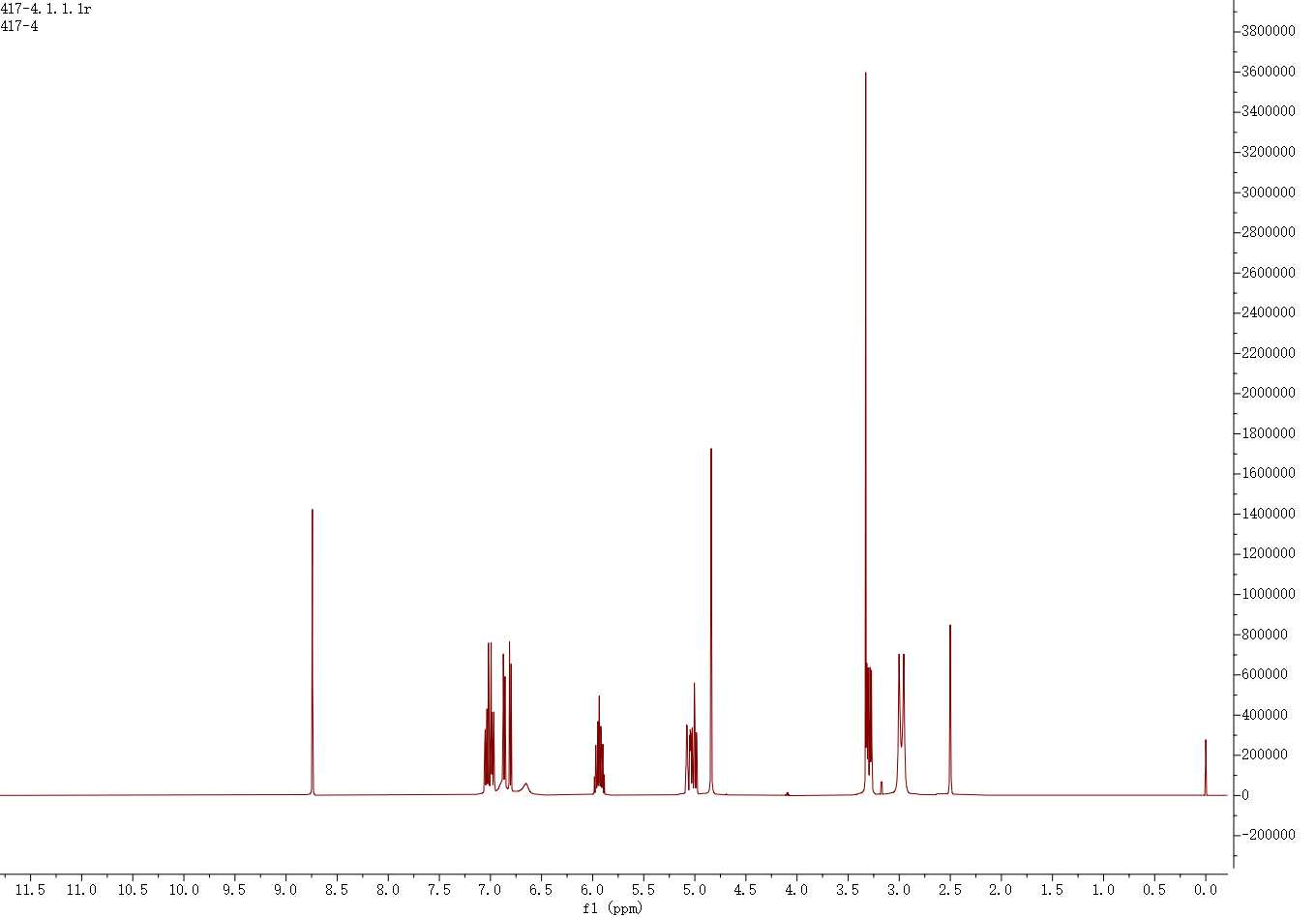

Supplement: Supplementary file 1 [file molecules-25-05779-s001.zip › SI/Compound1_1HNMR.jpg]

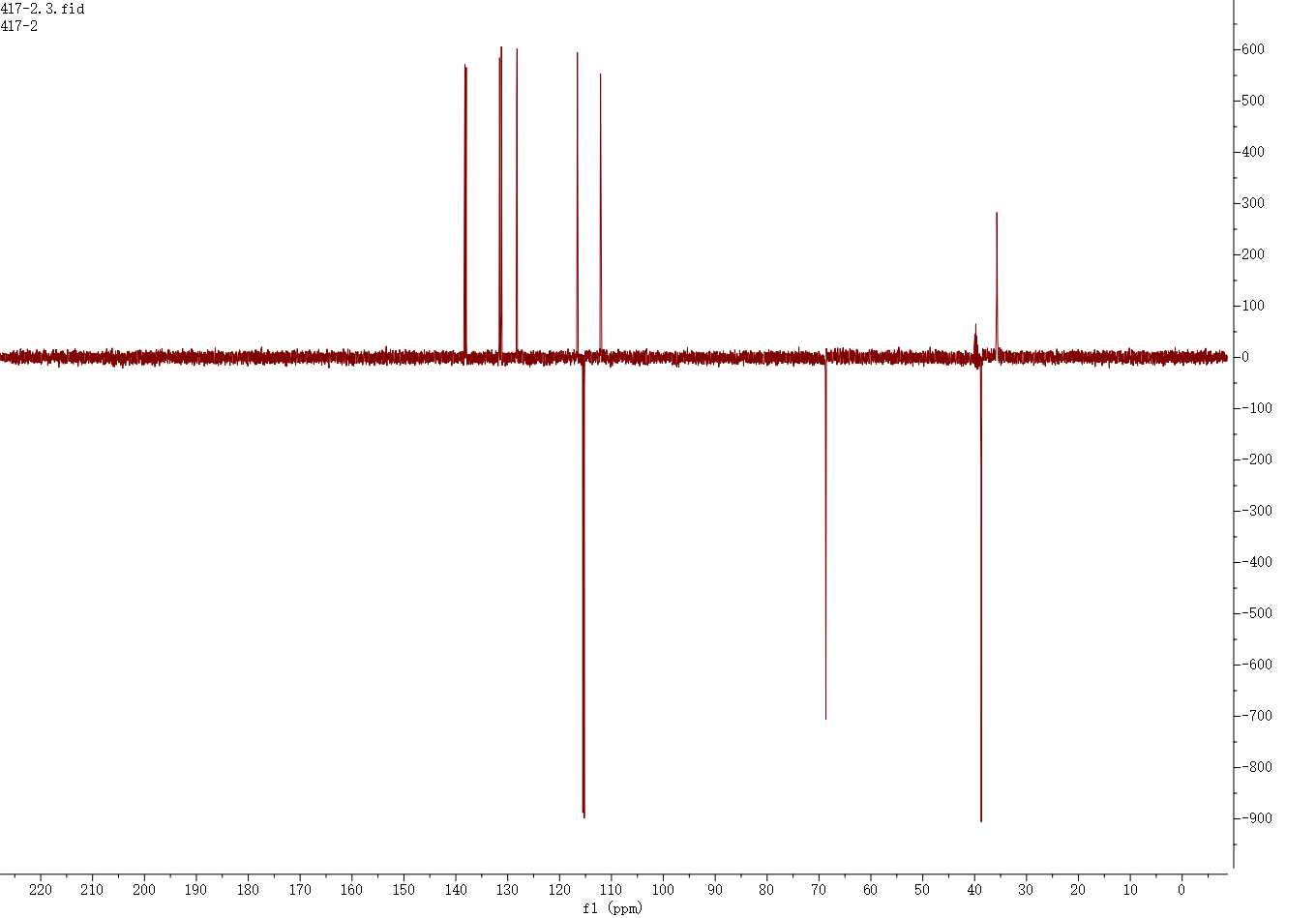

Supplement: Supplementary file 1 [file molecules-25-05779-s001.zip › SI/Compound1_DEPT.jpg]

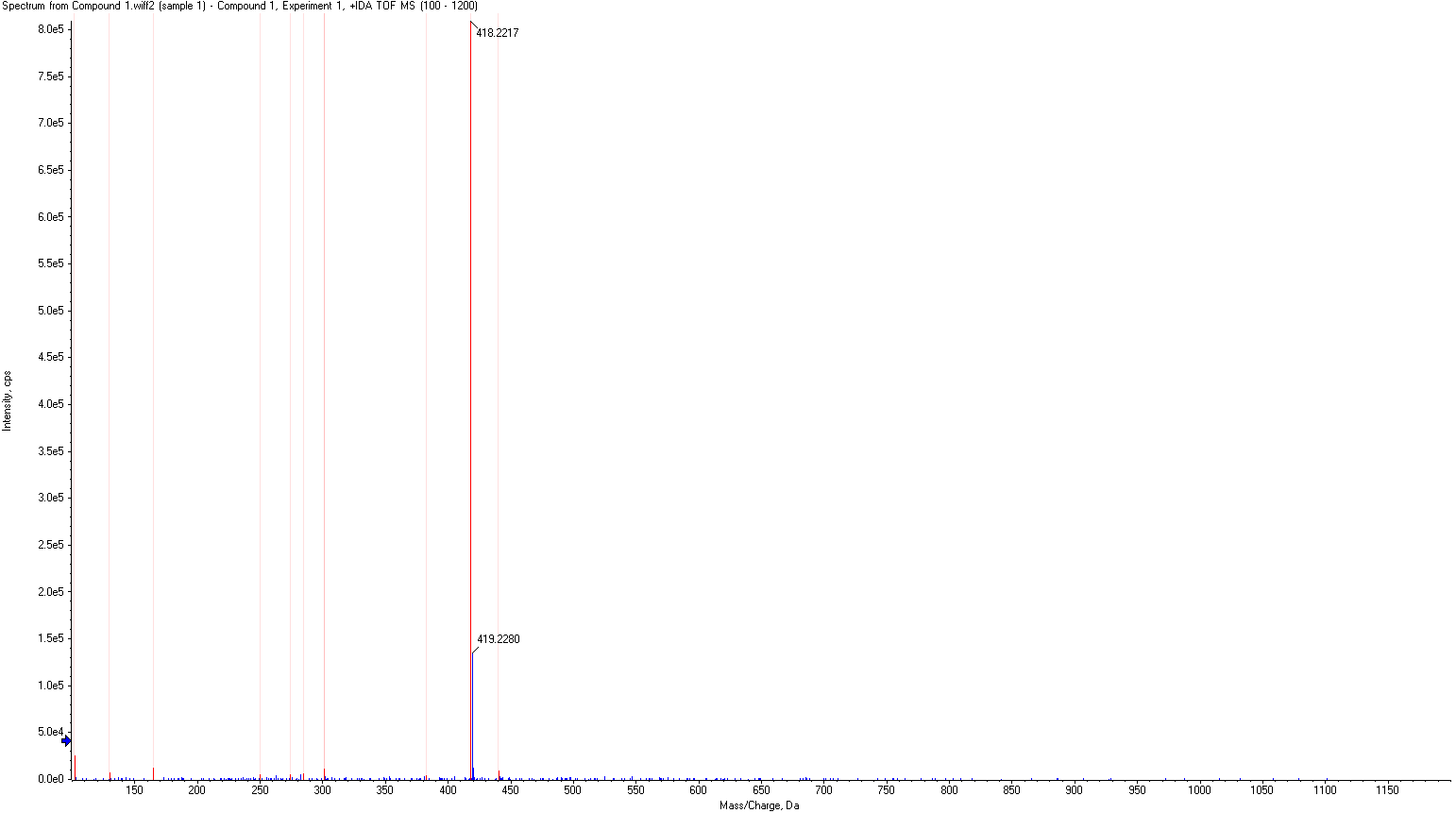

Supplement: Supplementary file 1 [file molecules-25-05779-s001.zip › SI/Compound1_MS.png]

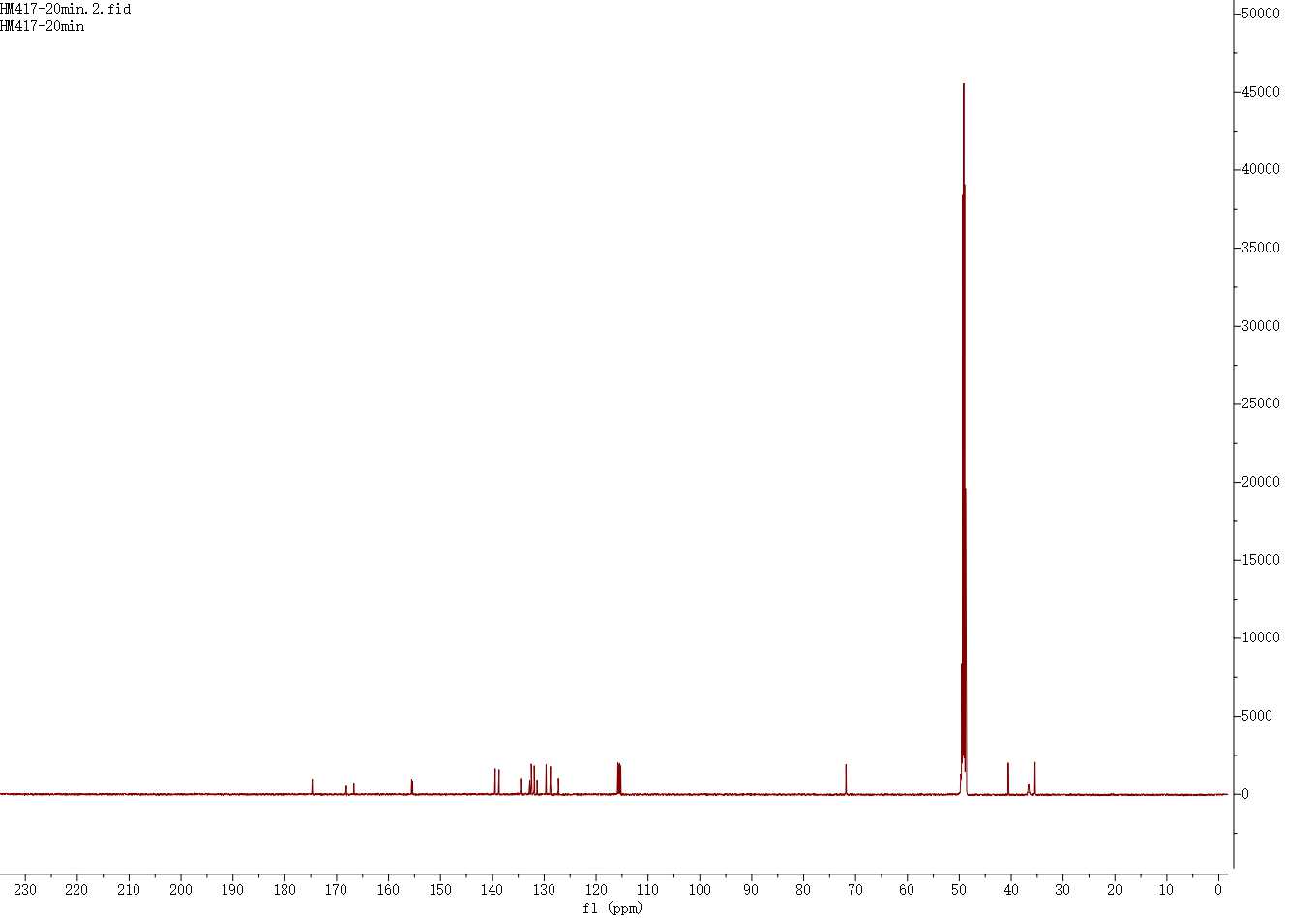

Supplement: Supplementary file 1 [file molecules-25-05779-s001.zip › SI/Compound2_13CNMR.jpg]

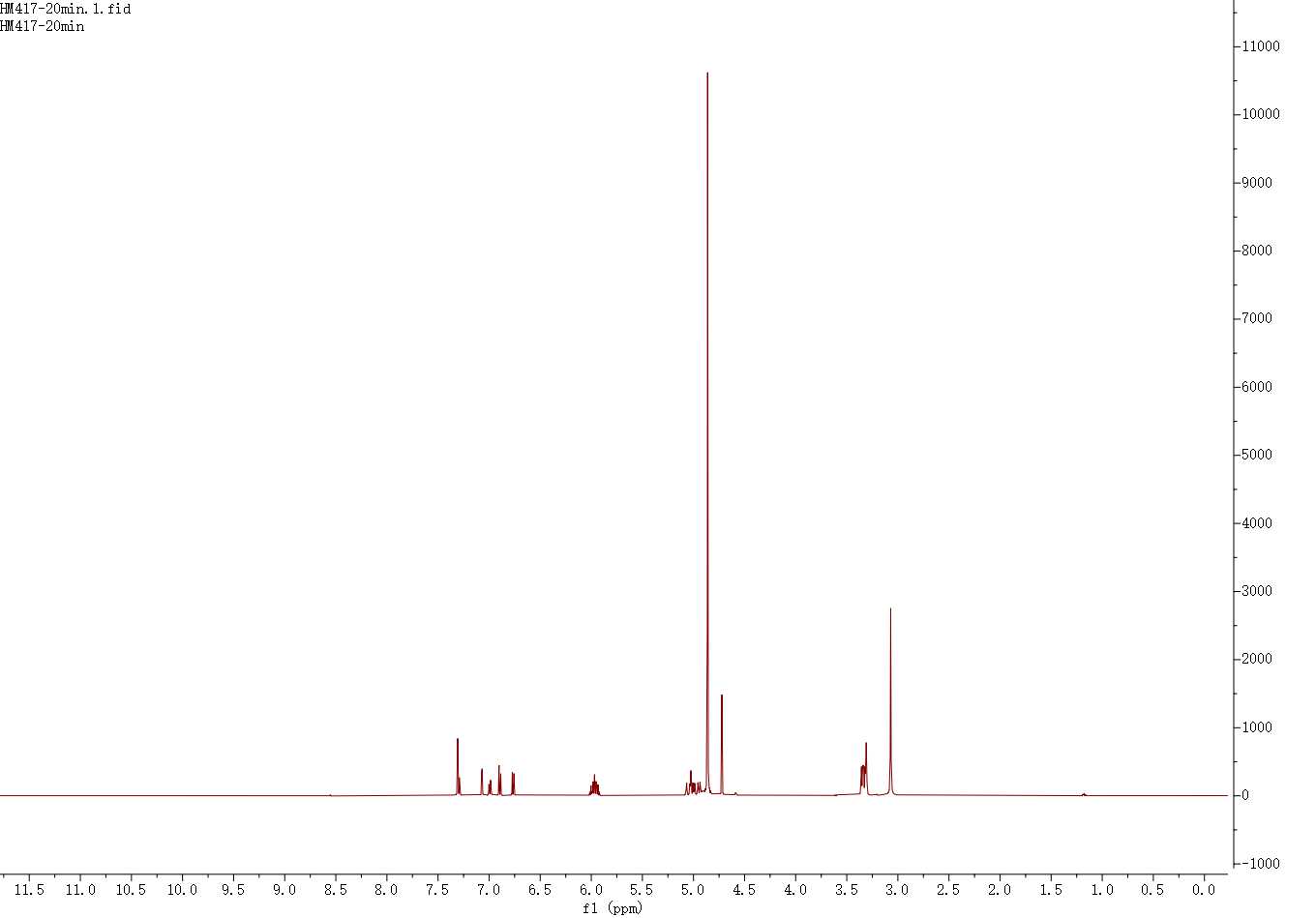

Supplement: Supplementary file 1 [file molecules-25-05779-s001.zip › SI/Compound2_1HNMR.jpg]

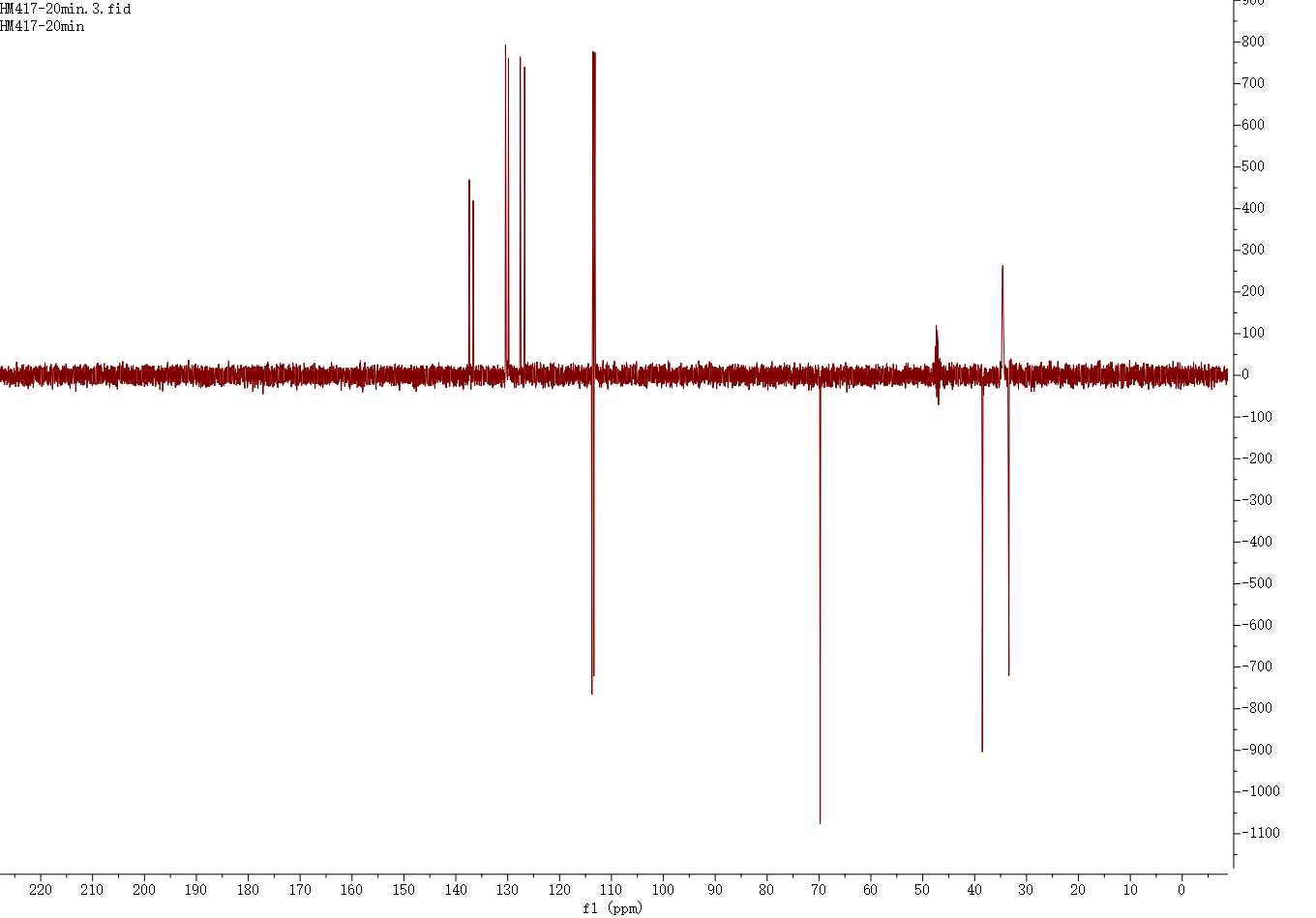

Supplement: Supplementary file 1 [file molecules-25-05779-s001.zip › SI/Compound2_DEPT.jpg]

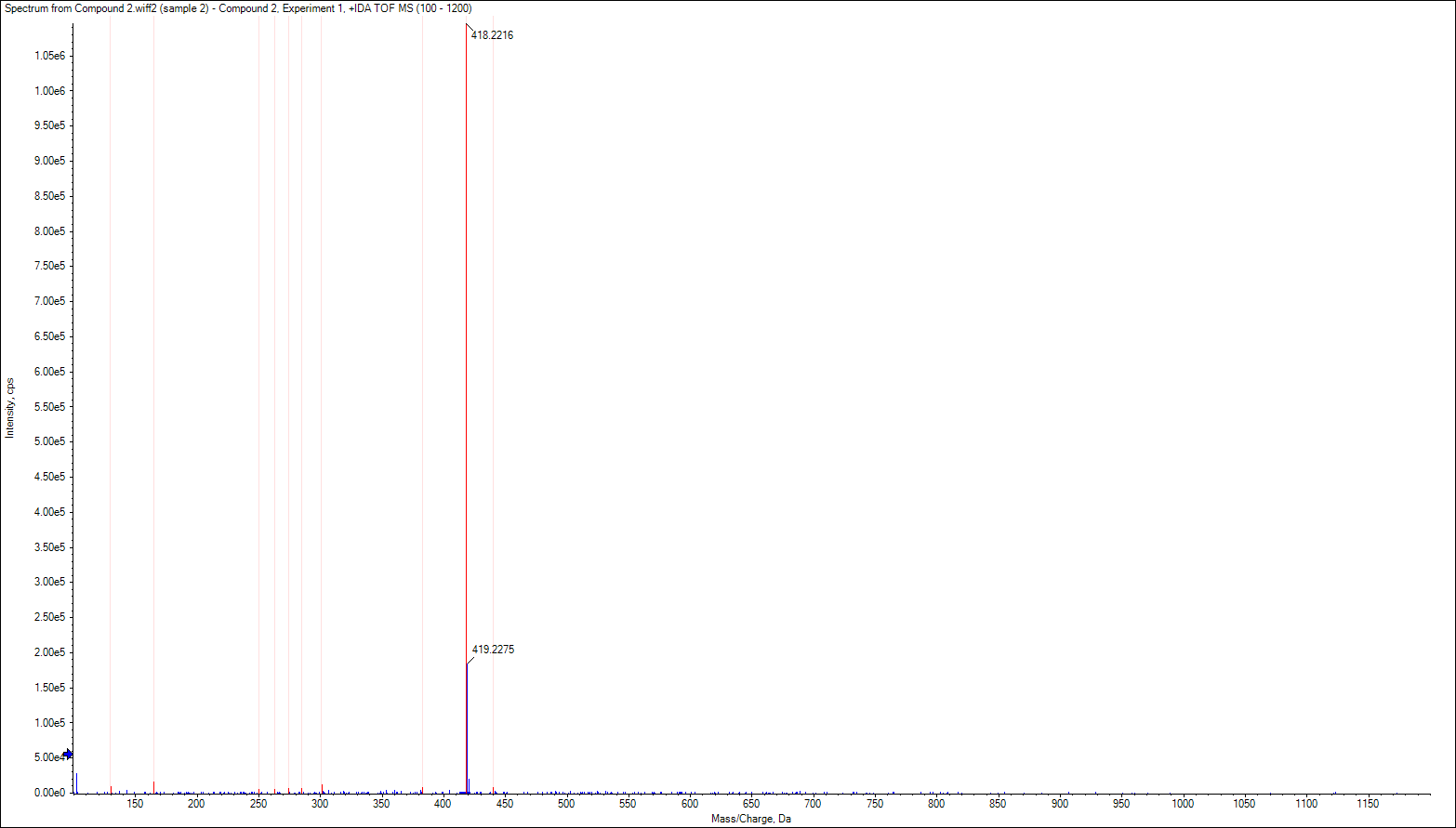

Supplement: Supplementary file 1 [file molecules-25-05779-s001.zip › SI/Compound2_MS.png]

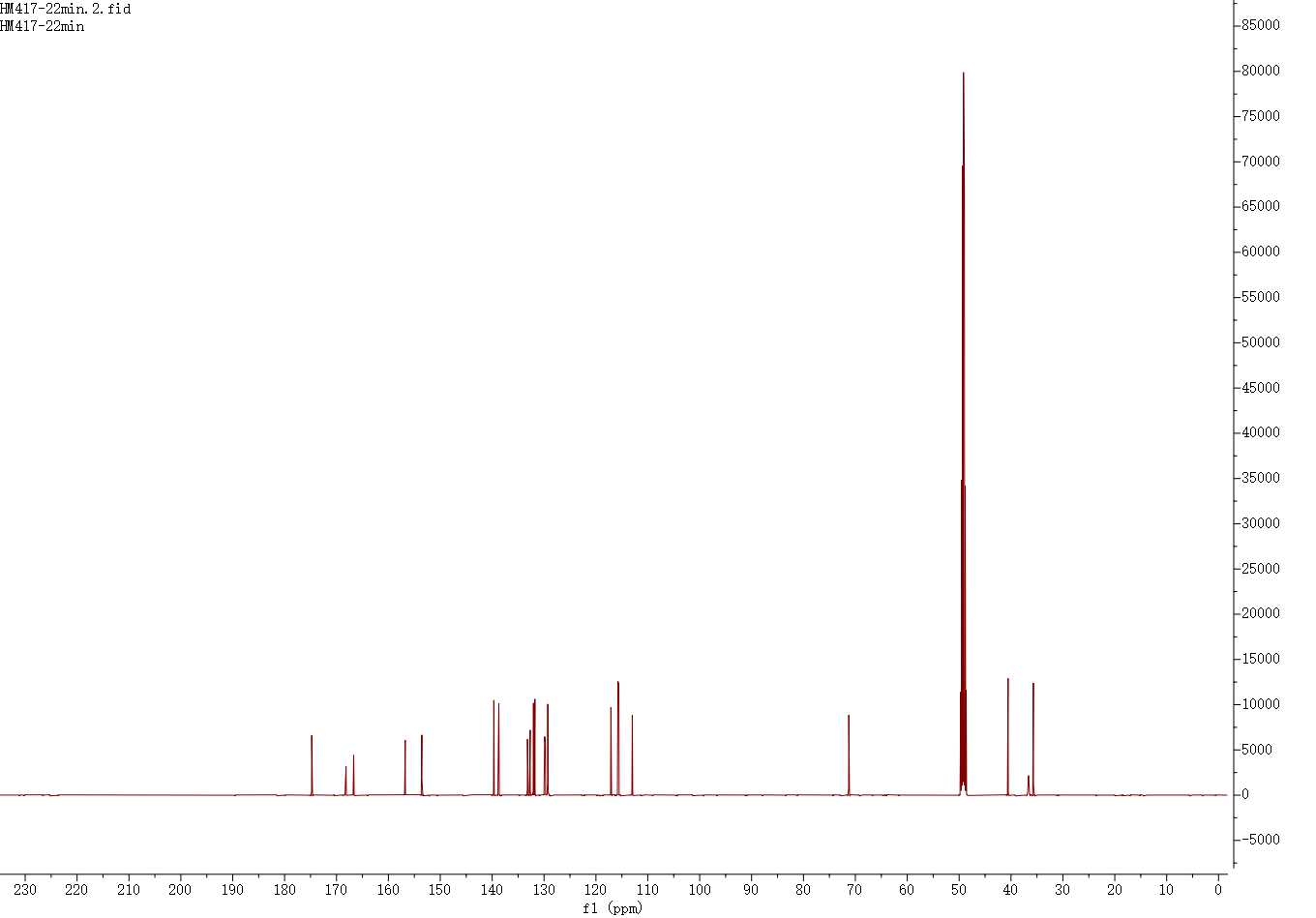

Supplement: Supplementary file 1 [file molecules-25-05779-s001.zip › SI/Compound3_13CNMR.jpg]

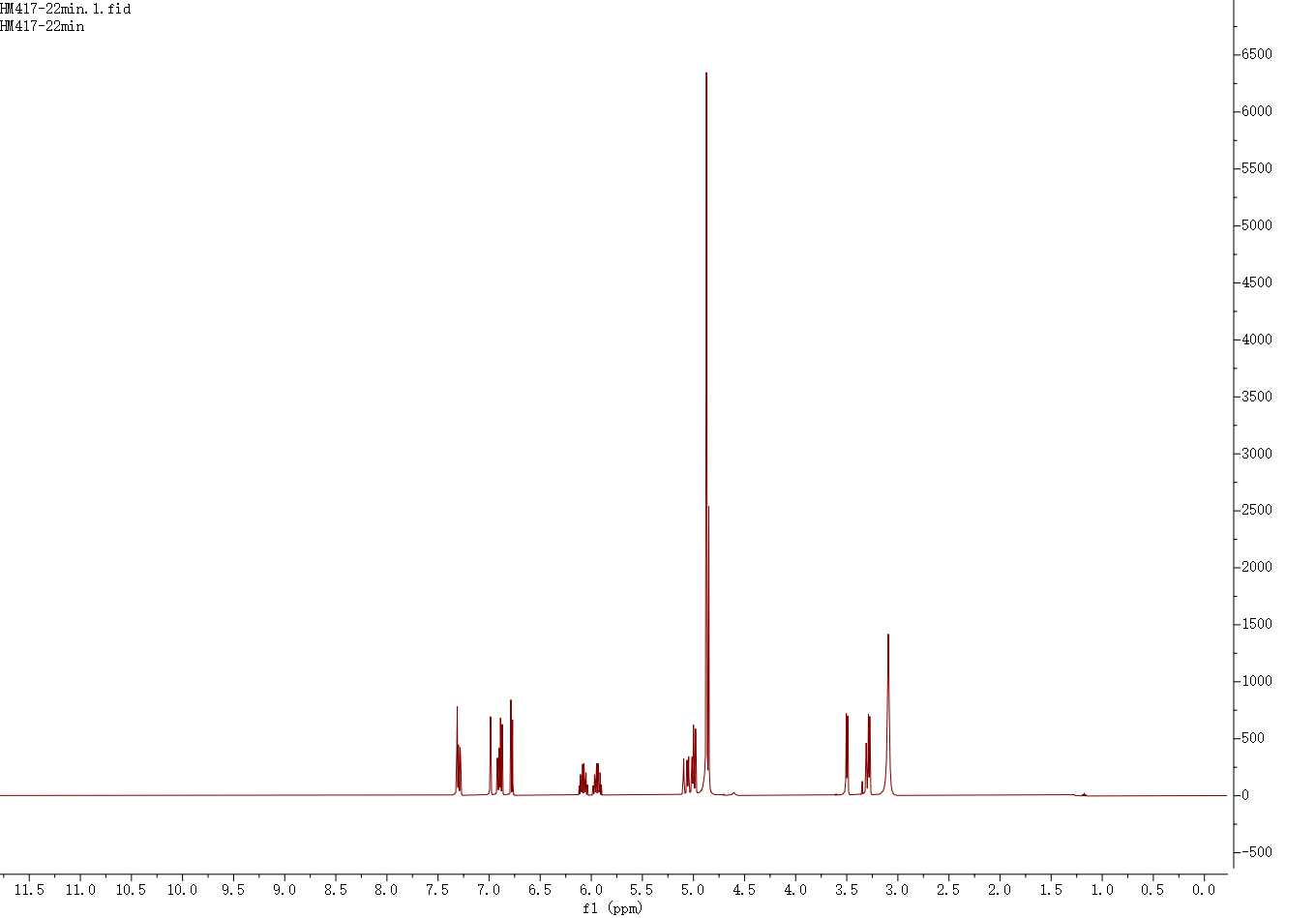

Supplement: Supplementary file 1 [file molecules-25-05779-s001.zip › SI/Compound3_1HNMR.jpg]

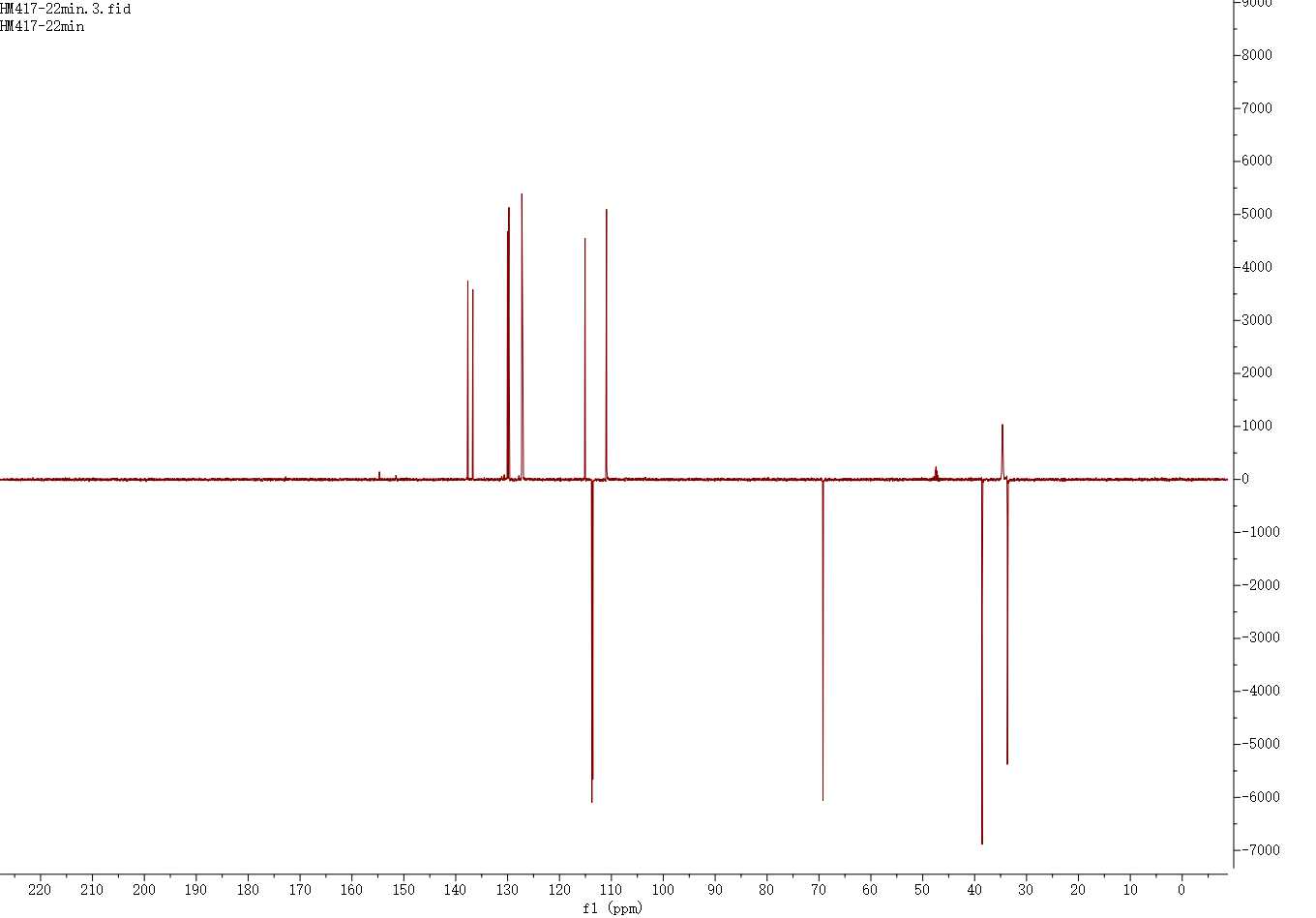

Supplement: Supplementary file 1 [file molecules-25-05779-s001.zip › SI/Compound3_DEPT.jpg]

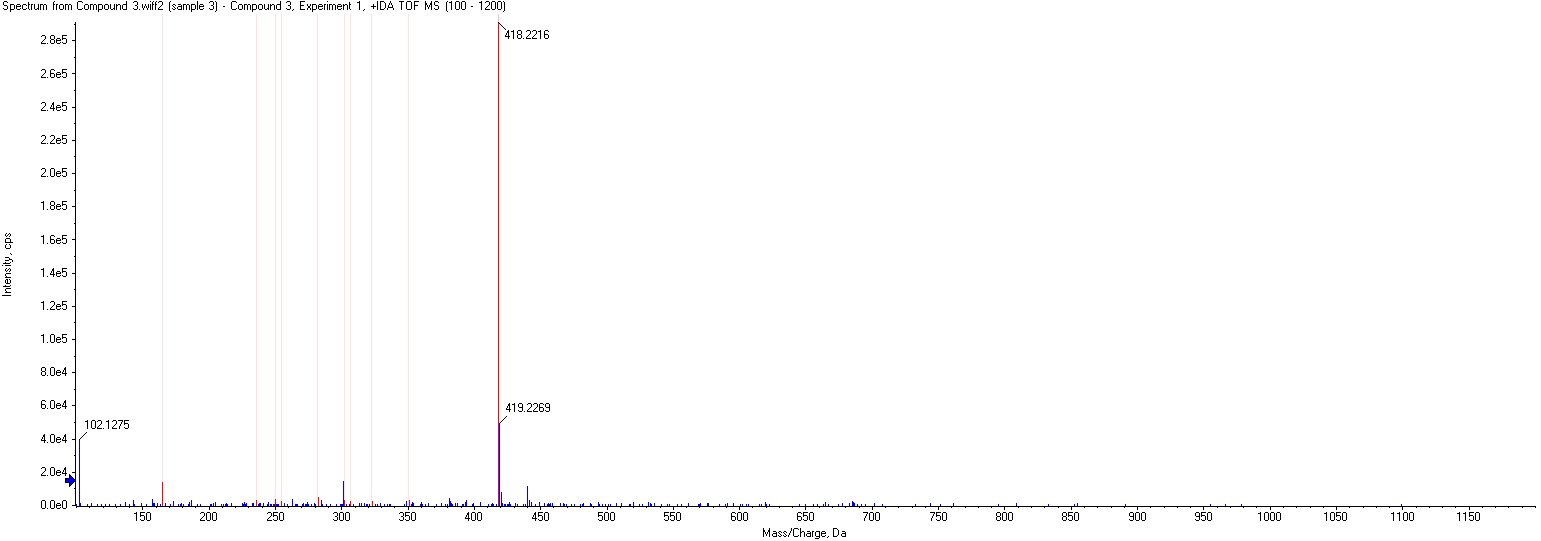

Supplement: Supplementary file 1 [file molecules-25-05779-s001.zip › SI/Compound3_MS.png]
